# Accounting for the aircraft emissions of China’s domestic routes during 2014–2019

**DOI:** 10.1038/s41597-022-01494-0

**Published:** 2022-07-06

**Authors:** Qiang Cui, Bin Chen, Yi-lin Lei

**Affiliations:** School of Economics and Management, Southeast University, Nanjing, 211189 China

**Keywords:** Environmental impact, Environmental impact, Governance

## Abstract

The “13th Five-Year Plan” of civil aviation energy conservation and emission reduction impacts China’s domestic aviation exchanges. However, few researchers pay attrition to the impact of the 13th Five-Year Plan on aviation emissions. This paper intends to calculate the emissions of six pollutants (CO_2_, CO, HC, NO_x_, PM2.5, and SO_2_) in China’s domestic airlines from 2014 to 2019 and explore the impact of the 13th Five-Year Plan on emissions changes. In this paper, the improved BFFM2-FOA-FPM method is used to unify the calculation of CO_2_ and other aviation emissions. The error rate between the estimate and the official data was about 6.45%. The results show that the 13th Five-Year Plan has impacted on aviation emissions, including the number of routes and airlines, aircraft configuration, air routes, and airline unit turnover emissions. Additionally, the 13th Five-Year Plan’s effect is not significant, and it does not promote the reduction of emissions on domestic routes.

## Introduction

The global demand for air transportation has grown in the past ten years, and China has become the second-largest air transportation market. The rapid growth of air traffic will bring some economic benefits, but it also impacts the environment1. To protect the environment and reduce the emission of aviation pollutants, the 13th Five-Year Plan for Civil Aviation Energy Conservation and Emission Reduction (13th Five-Year Plan) is proposed to promote aviation emission reduction. The overall goal of the 13th Five-Year Plan is that by 2020, the average energy consumption of unit transport turnover and carbon dioxide emission of civil aviation will drop by more than 4% over the five years compared with the 12th Five-Year Plan. In addition, the average energy consumption of unit passenger throughput of industrial transport airports will drop by more than 15% over the five years compared with the end of the 12th Five-Year Plan, and the garbage will be harmless. On the other hand, the sewage treatment rate of new airports will reach more than 90%. Previous scholars paid little attention to policies when studying the influencing factors of aviation carbon emissions. Considering the impact of the 13th Five-Year Plan on aviation emissions is an interesting part of this paper.

Aviation emissions mainly include carbon dioxide (CO_2_), carbon monoxide (CO), nitrogen oxide (NOx), sulfur dioxide (SO_2_), hydrocarbons (HC), and particulate matter (PM2.5)^1,2^. Reducing the emission of pollutants from aircraft has received widespread attention. In 2019, the International Civil Aviation Organization (ICAO) issued emission reduction targets, including reducing greenhouse gas emissions by 50 percent by 2050. The Intergovernmental Panel on Climate Change (IPCC) points out that carbon dioxide produced by human activities is the most significant contributor to the greenhouse effect^3^. The International Civil Aviation Organization (ICAO) uses standards and recommendations from various aspects of international civil aviation to calculate carbon emissions and uses a distance-based calculation method to estimate individual aviation emissions using existing data of different aircraft types^4^.

Some scholars separately measured the carbon emissions at the Landing and Take-Off (LTO) cycle or the Climbing/Cruising/Descending (CCD) stage in the existing studies. In contrast, others estimated the carbon emissions throughout the whole process. LTO phase carbon emissions are generally calculated using the International Civil Aviation Organization (ICAO) standard emission calculation model^5–10^. For the CCD emissions, Cui *et al*. used the modified fuel percentage method (MFPM) to calculate the carbon emission intensity of major airliners at different distances and established the database of 413 major airlines in China in 2018^11^. Cokorilo estimated the emission of aircraft in Belgrade Airport using the ICAO method^12^. Next, Zhou *et al*. set up three scenarios to forecast China’s aviation CO_2_ emissions^13^. Next, Yang *et al*. calculated CO_2_ emissions of all passenger flights to and from Shanghai from the bottom up and predicted the changing trend of CO_2_ emissions in the next five years^14^. Finally, Yu *et al*. evaluated the CO_2_ emissions of civil aviation from top to bottom based on the consumption of aviation kerosene^15^.

Other pollutants emitted by aviation mainly include CO, NO_x_, SO_2_, HC, and PM2.5. Many methods can calculate aviation pollutant emissions, including ICAO advanced method^8,11,16,17^, ICAO Complex method^18–20^, FOA3.0 method specially provided by ICAO for calculating particulate emission index^6,21^, American EPA method^5,22^, and European EMEP method^23–26^, etc.

However, the current methods separate calculations of CO_2_ emissions from five other emissions. In this paper, the improved BFFM2-FOA-FPM method can unify the analysis of carbon dioxide and other aircraft emissions in the CCD phase, improving existing research. Compared with other methods, the calculation method in this paper unifies the calculation of six emissions, which saves a lot of time and cost. Besides, few current studies have segmented emissions intensity calculations for the voyage. In this paper, the total distance is divided into eight sections every 500 km, and the emission intensity of six pollutants at different distances is calculated. Finally, existing studies rarely consider sub-series of a given aircraft. Therefore, this paper finds specific aircraft types. For example, A320 – 232, A320–214, and A320–216 are the sub-series of the A320 series.

**Fig. 1:**
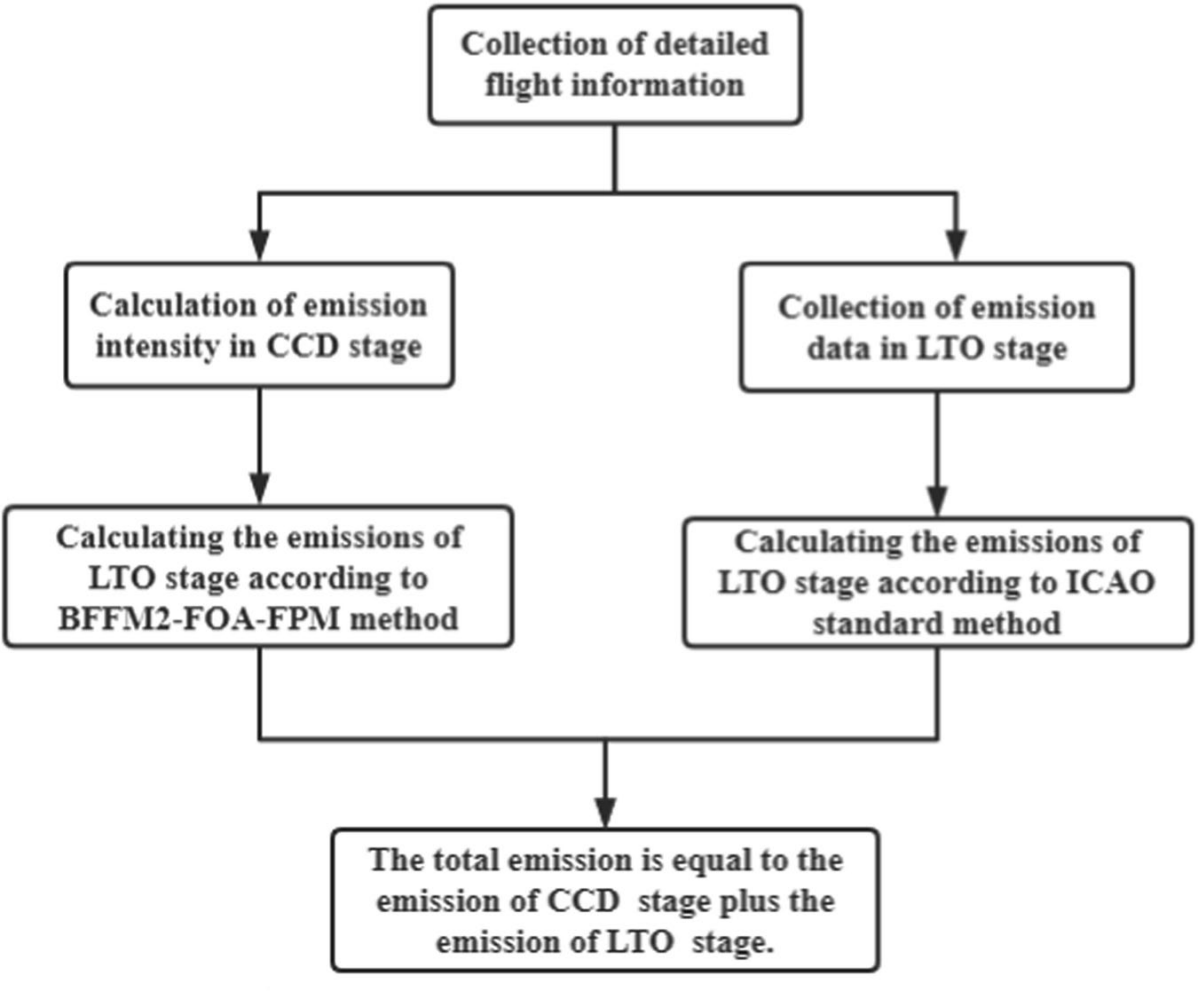
Research roadmap.

Through the research roadmap, we can quickly understand the research ideas of this paper. Fig. 1 is the research roadmap of this paper. This method is also applicable to other countries since the research steps can be copied. Of course, it is customary to encounter some difficulties in research. On the one hand, it is challenging to collect flight information. On the other hand, the error in different years may be substantial. We can reduce errors and improve the reliability of research results by extending the time axis.

## Results

This paper has not considered 2020 and 2021 because the pandemic began to spread globally in 2020. In addition, the outbreak of COVID-19 in December 2019 has had a significant impact on China’s aviation industry. As a result, China’s domestic routes completed a total transportation turnover of 58.767 billion ton-kilometers in 2020. Compared with 2019, China’s total domestic transportation turnover in 2020 decreased by 29.2%. Consequently, 2020 and 2021 are not selected in this paper. Therefore, the year from 2017 to 2019 was established after the 13th Five-Year Plan. Furthermore, 2014 to 2016 was chosen before the 13th Five-Year Plan for comparison. Finally, the total period is 2014–2019.

### Statistical characteristics of China’s domestic routes

**Fig. 2:**
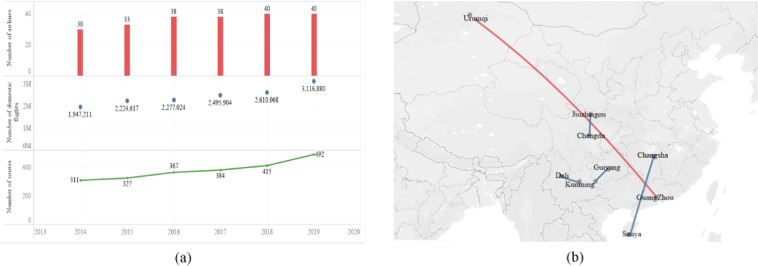
Statistical characters of the routes during 2014–2019. (**a**) Number of routes, number of airlines and number of domestic flights in China. (**b**) The farthest and nearest distance.

This paper gleans information on China’s domestic routes from 2014 to 2019, and the detailed statistical features are shown in Fig. 1. As shown in Fig. 2(a), the number of domestic flights in China has increased from 1,947,211 in 2014 to 3,116,880 in 2019, which shows the rapid growth of China’s domestic air transport demand. The number of routes has increased from 311 in 2014 to 492 in 2019, and the number of airlines has increased from 30 in 2014 to 40 in 2019. As shown in Fig. 2(b), from 2014 to 2019, the farthest flight route in China was always Guangzhou- Urumqi, and the distance was 3,836 km. However, the nearest routes within China had been in flux. The closest route in 2014 was Chengdu-Jiuzhai, with 350 km. From 2015 to 2017, Dali-Kunming became the nearest route with 275 km. In 2018, Changsha-Sanya (163 km) became the nearest route in China. In 2019, Xingyi-Guiyang became the nearest route in China, 305 km. Therefore, many airlines are constantly adjusting their routes to meet changing demands.

### The impacts of the 13th Five-Year Plan on aircraft configuration

**Fig. 3:**
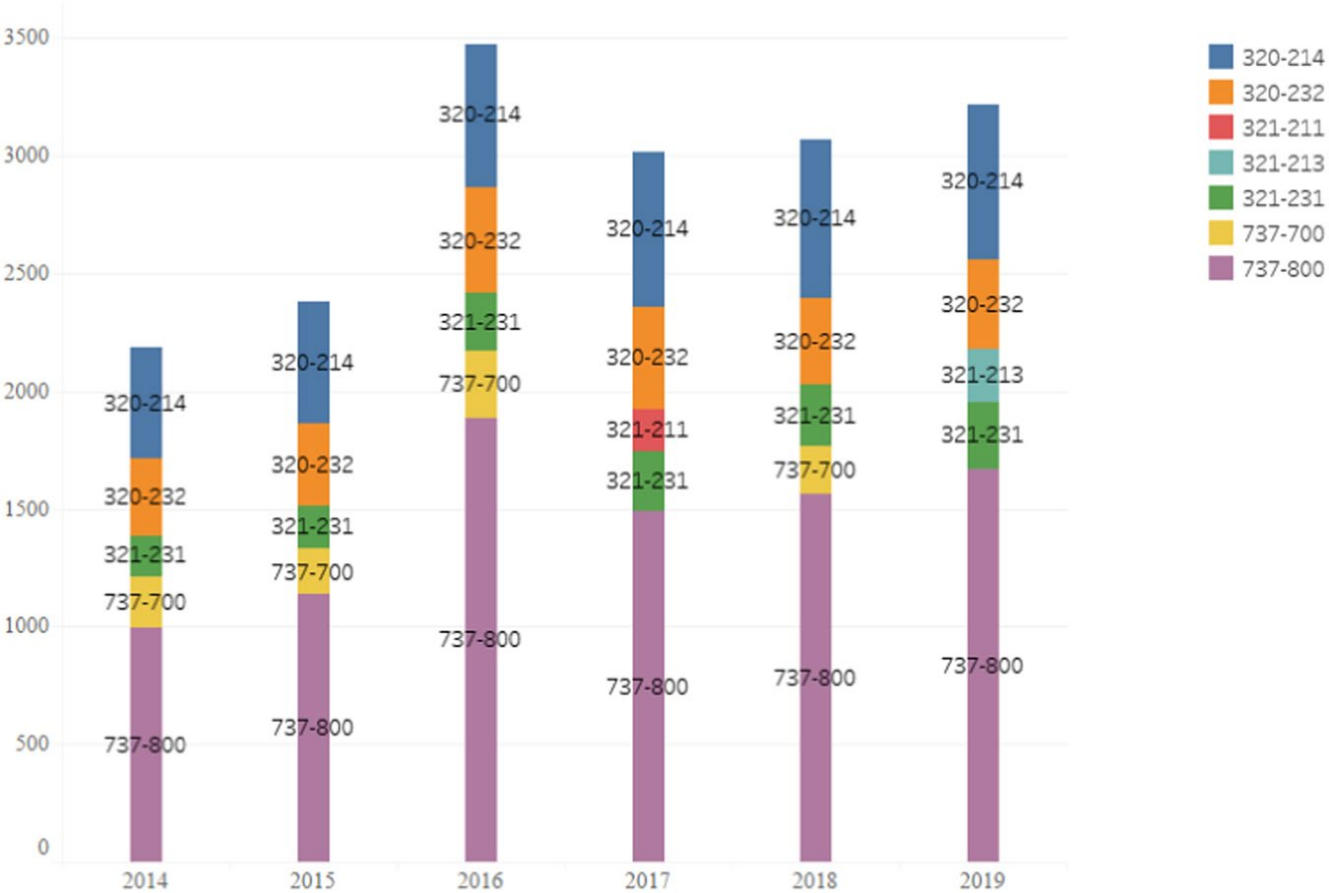
Statistical characteristics of aircraft configuration during 2014–2019.

This article makes detailed statistics on the configuration of aircraft types involved in China’s domestic routes from 2014 to 2019. First, the top five aircraft types for each year are shown in Fig. 3. After further study, we can find that the top three aircraft types have not changed from 2014 to 2019 which are 737–800, 320–214, and 320–232. In addition, the total frequency of 737–800 aircraft types has increased from 993 in 2014 to 1,669 in 2019; the entire frequency of 320–214 aircraft types has risen from 469 in 2014 to 660 in 2019; the total frequency of 320–232 aircraft types has increased from 332 in 2014 to 379 in 2019. However, the fourth and fifth-ranked aircraft types have changed with the 13th Five-Year Plan for Civil Aviation Energy Conservation Proposal and Emission Reduction Plan. Specifically, 737–700 and 321–231 ranked fourth and fifth respectively in 2014–2016, but 321–211 in 2017 and 231–213 in 2019 replaced the 737–700 and ranked fifth.

### The emission intensity of the aircraft in the CCD stage

As mentioned earlier, different from the method of ICAO, we segment each route according to a section of 500 kilometers. Therefore, all routes are divided into 8 distance sections: 0–500 km, 501–1000 km, 1001–1500 km, 1501–2000km, 2001–2500km, 2501–3000 km, 3501–3500 km and 3501–4000 km. In addition, we also consider the differences between sub-series, such as 737–700 and 737–800. Then, we get the aircraft’s emission intensity of the six pollutions from 2014 to 2019 based on the Modified Fuel Percentage Method (MFPM). In the 0–4000 km section, 320–214, 320–232, 737–700 and 737–800 cover almost all distances sections. The 320–214 and 320–232 are sub-series of the A320 series, while the 737–700 and 737–800 are sub-series of the B737 series. Therefore, this comparison highlights the difference between this study and the ICAO method.

**Fig. 4:**
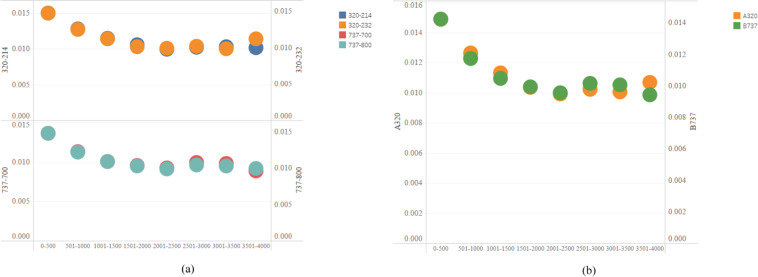
Carbon emission intensity of the aircrafts (ton/km). (**a**) Comparison of 320-214 and 320-232, and 737-700 and 737-800. (**b**) Comparison of A320 series and B737 series.

We summarize the average carbon emission intensity and show the detailed results in Fig. 4. As shown in Fig. 4(a), under the A320 series, carbon emission intensities of 320–214 and 320-232 are similar in the 0–4000 km distance segment. In the 3500–4000 km distance segment, the carbon emission intensity of 320-214 is lower than that of 320-232. However, at other distances, the carbon emission intensity of 320-232 is always less than 320-214. Therefore, 320-232 performs better in carbon emissions per kilometer, providing more references for airlines to arrange aircraft types. Under the B737 series, the carbon emission intensity of 737-700 and 737–800 is similar in 0–4000 km distance, and the 737–700 has always been less carbon-intensive than the 737–800. In addition, with the increase in aviation mileage, the emission intensity is gradually decreasing. This shows that the average emission of long-distance flights is less than that of short-range flights. Therefore, the 737-700 is superior to the 737–800 in the 0–4000 km distance segment. As shown in Fig. 4(b), compared to the A320 series, the B737 series has a lower carbon intensity. Hence, the overall performance of the B737 series at 0–4000 km is better than that of the A320 series.

### The impacts of the 13th Five-Year Plan on the overall emissions

First, we check the accuracy of the calculation results in this paper. Then, we obtain each airline’s available tonnage and normal load rate from the “Civil Aviation from Statistics” released by CAAC (Civil Aviation Administration of China). The turnover in Table 1 is the total turnover of China’s domestic routes, and the fuel consumption per ton kilometer is the common data of China’s domestic routes and China’s foreign routes. The turnover of each route is the product of available tonnage, normal load rate, and distance. Then we can add up the turnover of all routes. In 2015, the fuel consumption of turnover was about 0.294 kg/ ton. Because of the error, the fuel consumption of turnover can be adjusted by 5%, and the result after adjustment is 0.3076 kg/ t-km. Based on this standard multiplied by the carbon emission coefficient of fuel consumption (3.157 kg/tons), it can be concluded that the carbon dioxide emission of major domestic routes in 2015 was 37,513,993.70 tons. Therefore, the carbon dioxide emissions calculated in this paper are 39,905,708.71 tons, with an error rate of 6.37%. Based on the above calculation method, the calculation errors from 2016 to 2019 are 0.75%, 0.9%, 15.05%, and 9.2%. Considering the statistical data of various airlines may also have errors, the calculation results of the method used in this paper are very accurate.

**Table 1:** The aviation emissions released by the CAAC from 2014 to 2019.

Year	Turnover(million ton kilometer)	Fuel consumption per ton kilometer(ton/ kilometer)
2014	50,808	—
2015	55,904	0.294
2016	62,193	0.293
2017	69,460	0.293
2018	77,151	0.287
2019	82,951	0.285
2020	58,767	0.316
2021	64,110	—

**Fig. 5:**
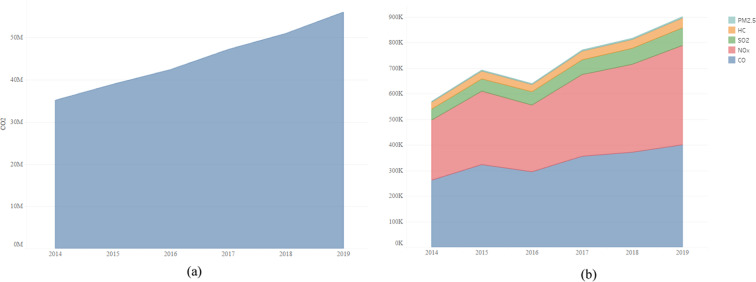
Changes in total emissions of six pollutants from 2014–2019. (**a**) Annual change in carbon dioxide emissions from 2014 to 2019. (**b**) Annual changes in CO, HC, NO_x_, PM2.5 and SO_2_ emissions from 2014 to 2019.

As mentioned earlier, the main emissions include CO_2_, CO, HC, NOx, PM2.5, and SO_2_, among which CO_2_ emissions are much higher than other emissions. For example, in 2019, CO_2_ emissions is 56,059,114.91 tons, accounting for 98.42% of the total emissions(56,960,300.60 tons); Emissions of CO, HC, NOx, PM2.5 and SO_2_ are 400,395.30 tons, 37,998.9572 tons, 388,677.70 tons, 5,393.817,46 tons and 68,719.9160 tons, which account for 0.7%, 0.07%, 0.68%, 0.01% and 0.12% of total emissions (see details in supplementary files). Since the 13th Five-Year Plan was put forward in February 2017, the data are divided into two groups. One is 2014–2019 (before the 13th Five-Year Plan), and the other is 2017–2019 (After the 13th Five-Year Plan). Fig. 5 is a comparison of the average emissions from 2014 to 2019. As shown in Fig. 5, after the 13th Five-Year Plan, the six emissions increased, but the growth rate was less than 35%. Further research finds that the growth rate of NOx is the highest, reaching 34.5%. HC has the lowest growth rate at 23.7%. In terms of the annual growth rates of the six emissions, the growth rates of 2015 and 2016 are higher than those of 2018 and 2019, which may be due to the 13th Five-Year Plan that urges airlines to take some measures to reduce the growth rate of aviation emissions.

**Fig. 6:**
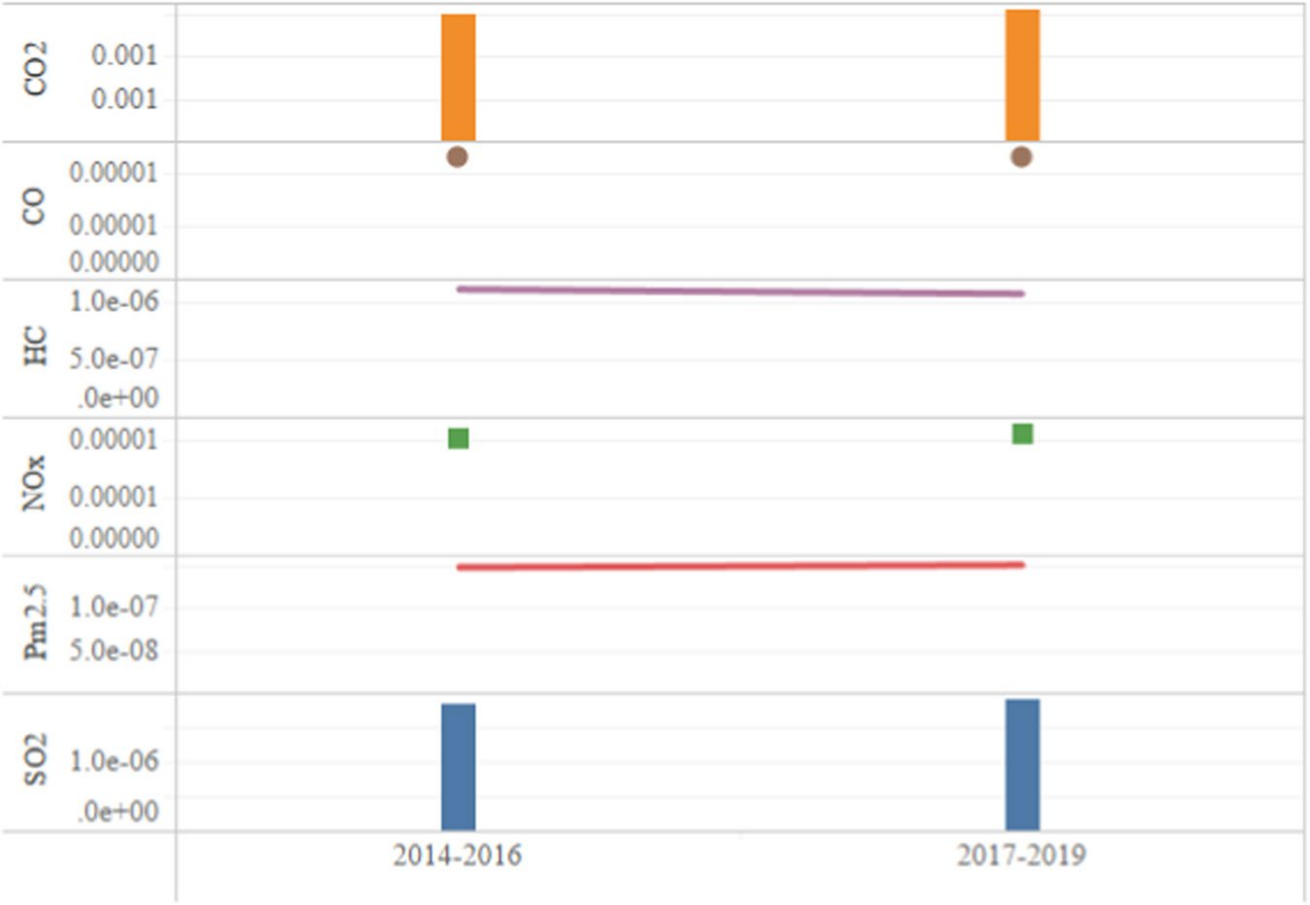
Unit turnover emissions in 2014–2016 and 2017–2019.

In addition, we compared the change in unit turnover emissions of 6 pollutants before and after the 13th Five-Year Plan. According to the calculation of relevant report data from the CAAC, the total annual transport turnover from 2014 to 2019 was 34.88 billion ton-kilometers, 38.62 billion ton-kilometers, and 43.36 billion ton-kilometers, 48.16 billion ton-kilometers, 46.58 billion ton-kilometers, and 54.34 billion ton-kilometers. And then, unit turnover emissions of six gases from 2014 to 2019 can be calculated. The average results of the first three years and the last three years are shown in Fig. 6. The unit turnover emissions of HC decreased, while the unit turnover emissions of the other five gases all increased. Therefore, the 13th Five-Year Plan produces a miniature effect, and China’s domestic airline emission reduction work still needs improvement.

### The impacts of the 13th Five-Year Plan on the emissions of the routes

We firstly analyze the effect of the 13th Five-Year Plan on the average emissions of airlines. Compared with 2014–2016, the average CO2, NOx, PM2.5, and SO2 in 2017–2019 increased by 2.58%, 4.67%, 1.61%, and 3.45%. In addition, the average emissions of CO and HC decreased by 0.03% and 3.57%. As a result, the average CO2, NOx, PM2.5, and SO2 increased by less than 5%, while the average emissions of CO and HC decreased. As a result, the average emissions growth rate of the six air routes in 2018 and 2019 was negative. Therefore, the 13th Five-Year Plan has slowed down the increase rate of airline emissions.

To investigate the influence of the 13th Five-Year Plan on air routes, we select 251 airlines from 2014 to 2019. However, we split the route data into two groups: 2014–2016 and 2017–2019. Besides, the paper calculates the average unit turnover carbon emissions of two data groups for 251 airlines. The calculation result shows that the average unit turnover carbon emissions of 126 airlines are reduced.

**Fig. 7:**
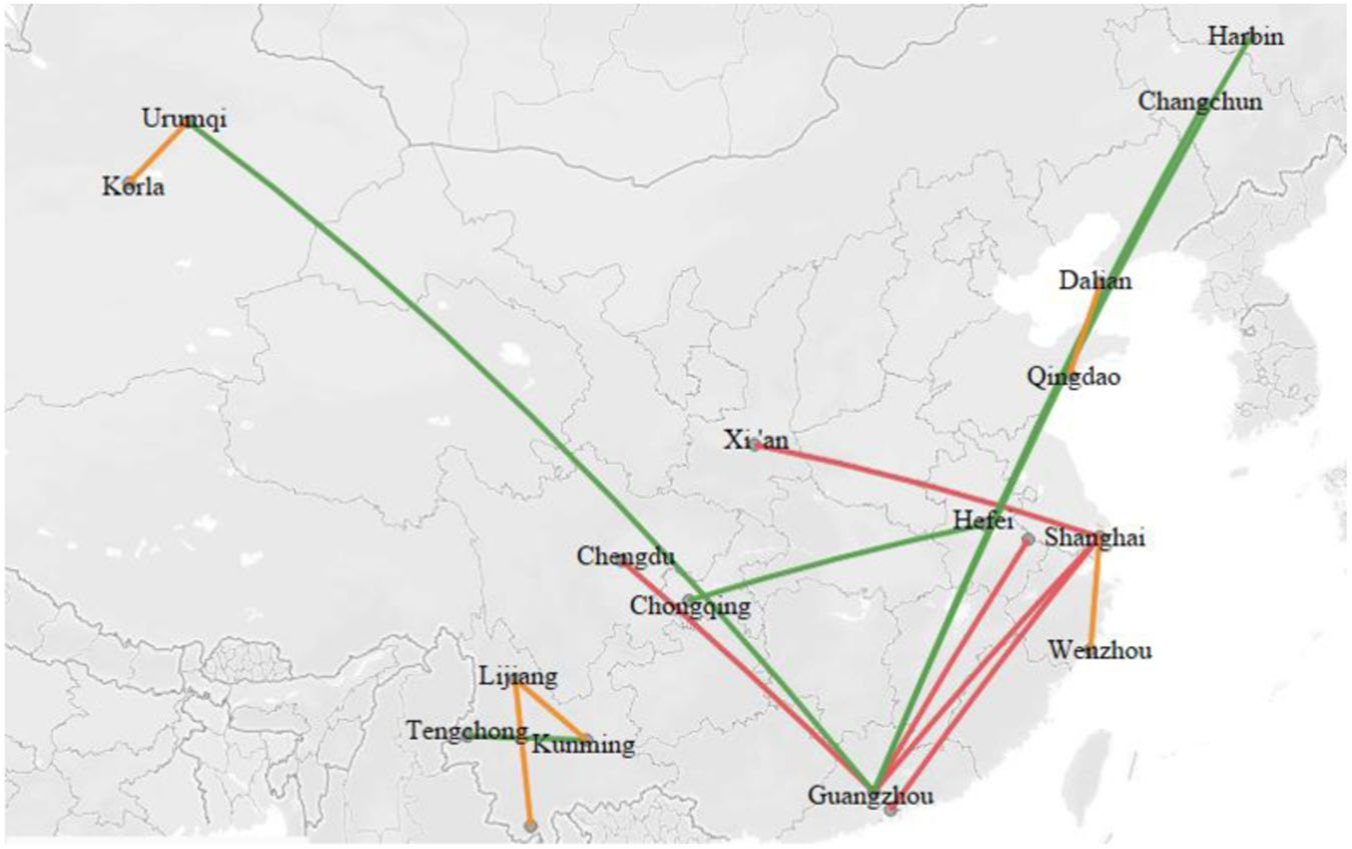
Influence of the 13th Five-Year Plan on some popular routes.

We summarize the impact of the 13th Five-Year Plan on unit turnover emissions of some popular air routes and show the detailed result in Fig. 7. As shown in Fig. 7, this paper picks out the five airlines with the most significant increase and the five airlines with the largest decrease in unit turnover emissions under the influence of the 13th Five-Year Plan. Specifically speaking, the five routes with the most significant increase in unit turnover carbon emissions are Korla-Urumqi, Dalian-Qingdao, Kunming-Lijiang, Shanghai-Wenzhou, and Xishuangbanna-Lijiang. As a result, the average unit turnover carbon emissions of these five airlines increased by 2.86E-02 tons, 2.66E-02 tons, 2.32E-02 tons, 2.04E-02 tons, and1.02E-02 tons. Moreover, the five airlines with the most considerable reduction in unit turnover carbon emissions are Guangzhou-Harbin, Guangzhou-Changchun, Guangzhou-Urumqi, Chongqing-Hefei, and Kunming-Tengchong. As a result, the average unit turnover carbon emissions of these five airlines decreased by 1.10E-03 tons, 1.31E-03 tons, 1.33E-03 tons, 1.71E-03 tons, and 2.06E-03 tons. On the other hand, the five routes least affected by the 13th Five-Year Plan are Guangzhou-Nanjing, Guangzhou-Shanghai, Shanghai-Xi’an, Shanghai-Shenzhen, and Guangzhou-Chengdu. More concretely, the average unit turnover carbon emissions of Guangzhou-Nanjing, Guangzhou- Shanghai, and Shanghai-Xi’an increased by 5.83E-08 tons, 2.92E-07 tons, and 1.06E-06 tons. On the other hand, the average unit turnover carbon emissions of Shanghai-Shenzhen and Guangzhou-Chengdu decreased by 3.37E-06 tons and 3.61E-06 tons.

### The impacts of the 13th Five-Year Plan on the average emissions of airlines

Compared with 2014–2016, the average emissions of CO_2_, CO, HC, NOx, PM2.5 and SO_2_ in 2017–2019 increased by 12.03%, 8.95%, 5.05%, 14.36%, 10.81% and 12.98%. The average emission growth rate of CO_2_, CO, HC, NOx, PM2.5, and SO_2_ is 5%-15%. The average emissions of all six air routes showed a higher growth rate in 2017, which may be because there was no increase in the number of airlines in 2017 (38 in both 2016 and 2017), and the emissions of the six air routes in 2017 were higher than those in 2016.

**Fig. 8:**
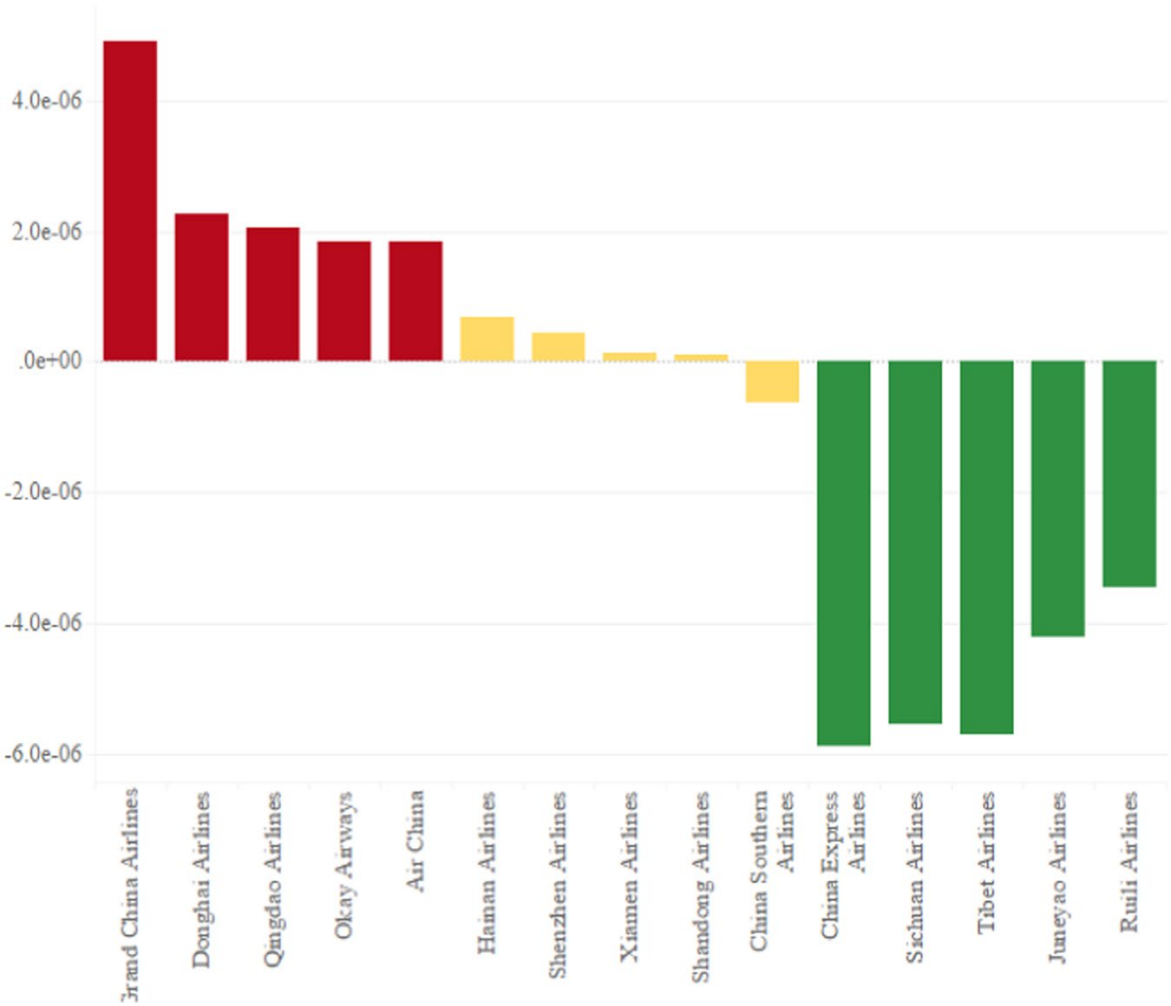
Influence of the 13th Five-Year Plan on some popular airlines.

To analyze the impacts of the 13th Five-Year Plan on airlines, this paper selected 27 standard airlines from 2014 to 2019. In addition, airline data is divided into two groups, one is 2014–2016, and the other is 2017–2019. And then, the paper calculates the average unit turnover carbon emissions of two data sets from 251 airlines. The calculation result shows that the carbon emissions per unit turnover of 16 airlines have been reduced. Finally, we summarize the change in carbon emissions per unit turnover of popular airlines before and after the 13th Five-Year Plan in Fig. 8. As shown in Fig. 8, the five airlines with the most significant increase in carbon emissions unit turnover were Grand China Airlines, Donghai Airlines, Qingdao Airlines, Okay Airways, and Air China. As a result, the five airlines’ average unit turnover carbon emissions increased by 4.91E-06tons, 2.25E-06tons, 2.05E-06tons, 1.85E-06tons, and 1.84E-06tons. The five airlines with the most significant drop in unit turnover carbon emissions are Ruili Airlines, Juneyao Airlines, Sichuan Airlines, Tibet Airlines, and China Express Airlines, respectively, which decreased by 3.45E-06 tons, 4.20E-06 tons, 5.56E-06 tons, 5.72E-06 tons, and 5.89E-06 tons. The five airlines least affected by the 13th Five-Year Plan of Civil Aviation Energy conservation and emission reduction are Shandong Airlines, Xiamen Airlines, Shenzhen Airlines, China Southern Airlines, and Hainan Airlines. Among these airlines, the unit turnover carbon emissions of Shandong Airlines, Xiamen Airlines, Shenzhen Airlines, and Hainan Airlines increased by 8.81E-08 tons, 1.39E-07 tons, 4.26E-07 tons, and 6.84E-07 tons. In contrast, China Southern Airlines’ unit turnover carbon emissions decreased by 6.14E-07 tons.

## Discussion

This study shows solicitude for the impact of the 13th Five-Year Plan on aircraft emissions of major China domestic routes in 2014–2019. Since the 13th Five-Year Plan was put forward in February 2017, 2014–2016 is the data before the 13th Five-Year Plan, and 2017–2019 was the data under the 13th Five-Year Plan. First, we collect flight information on major domestic routes in China, including aircraft type, flight frequency, route, flight distance, flight time, etc. And then, we calculate the total emissions for each route and airline containing CO_2_, CO, HC, NOx, PM2.5, and SO_2_. Overall emissions include CCD emissions and LTO emissions. CCD stage is calculated by the Modified BFFM2-FOA-FPM method, LTO stage is calculated by ICAO standard method. The emission accounting can better summarize the impact of aircraft activities on the environment and provide data and method references for putting forward corresponding countermeasures.

The main contribution of this paper to the literature is reflected in the following aspects.

In the first place, it is the attempt to combine calculations of carbon dioxide emissions with other aircraft emissions. Some papers focus on calculating CO_2_ and other aircraft emissions, but there is no uniform way to link the two accounting methods. In addition, the calculation method of CO_2_ emission does not consider the difference of sub-series, and the calculation method of other aircraft emissions also does not pay attention to the emission of the CCD stage. Therefore, we establish a new Modified BFFM2-FOA-FPM method to calculate CO_2_ and other aircraft emissions in the CCD stage. LTO emissions are calculated according to the International Civil Aviation Organization (ICAO). The error rate between the computed results and the official data is about 6.45%. This paper applies this method to domestic airlines in China, but it can also be used in other countries or regions.

Secondly, the emission intensity of aircraft types has been calculated first. To ensure the accuracy of the calculation results, we divided the air route distances into eight groups to obtain the emission intensity of 6 pollutants of aircraft types at different distances. As a result, our results cover more detailed aircraft types (such as 320–214, 320-232, and 320-216 under the A320 series) than existing studies, making them more realistic.

The main conclusions and policy recommendations are as follows. First, this paper analyzes the impact of the 13th Five-Year Plan on aircraft configuration. There is no change in the top three (737–800, 320-214, and 320-232) from 2014 to 2019. However, the fourth and fifth places adjusted over time, and 737-700, 321-231, 321-211, and 321-213 appeared in the fourth or fifth places. Second, this paper compares the emission intensity of 6 kinds of aviation emissions in the CCD stage. We consider more detailed aircraft types, which is one of the contributions of this paper. We compared the sub-series of the B737 series with those of the A320 series. We found the aircraft types with the lowest emission intensity of the B737 series and the A320 series sub-series, providing a reference for airlines to adjust aircraft configuration. Third, we discover CO_2_ always accounts for more than 98% of the total emissions, while the emissions of the other five gases are relatively small. Except for the decrease in HC unit turnover, the additional five gas unit turnover shows an upward trend, indicating that the 13th Five-Year Plan has little impact on domestic aviation. Finally, there were 251 same air routes and 27 same airlines from 2014 to 2019. Under the influence of the 13th Five-Year Plan, 127 air routes and 16 airlines have reduced unit turnover carbon emissions, which shows that the 13th Five-Year Plan impacts the emissions of domestic flights, but this impact is not particularly obvious.

There are technical approaches, market approaches, and management approaches to control aircraft emissions. The technical method mainly involves improving the engine, increasing its fuel efficiency, and using sustainable aviation fuel. The market approaches mainly include aviation pollution tax and carbon emission trading. Calculating carbon dioxide and other aviation emissions is the basis and premise of imposing pollution tax and trading carbon emission rights. Scientific management is mainly reflected in airlines to strengthen the efficiency of air traffic control operation organization and guarantee ability. As mentioned above, the emission intensity of aircraft in different flight segments is different, and the emission intensity decreases with the increase in flight distance. Therefore, airlines can optimize route structure and develop long-distance routes. Thus, the work of this paper is of great significance.

Due to the availability of the data, the specific data analysis in this paper is based on major domestic airlines in China, which is incomplete. Additionally, the factors considered in this paper are not comprehensive enough, such as the age of the aircraft, navigation technology, and aircraft delay caused by various factors, which may have an impact on the emission intensity of the aircraft. Therefore, we can invest more time and energy to obtain more complete data and expand the research scope in future studies.

## Methods

Generally, the entire flight process consists of seven steps: Engine Starting, Taxiing, Taking Off, Climbing, Cruising, Descending, and Landing^27^. It is usually divided into the Landing and Take-Off (LTO) cycle and the Climbing/Cruising/Descending (CCD) stage. Therefore, the overall emissions include LTO emissions and CCD emissions. The emissions in the CCD stage are calculated through the Modified BFFM2-FOA-FPM method. The LTO emissions are calculated based on the ICAO standard method.

### Modified BFFM2-FOA-FPM method

Based on the modified Fuel Percentage Method (MFPM) method, we build a new method the Modified BFFM2-FOA-FPM method to combine the calculation of CO_2_ and non-CO_2_ emissions. In the Modified BFFM2-FOA-FPM method, the CCD emissions $E(Q)$ can be calculated by

1 $$\begin{array}{ccc}{E}_{j}(Q)={I}_{j}\times F(Q) & = & {I}_{j}\times {M}_{fuel}\times weight(Q)={I}_{j}\times (1-{M}_{ff})\times weight(Q)\\  & = & {I}_{j}\times \left(1-\mathop{\prod }\limits_{i=1}^{n}\frac{{W}_{i}}{{W}_{i-1}}\right)\times weight(Q)={I}_{j}\times \left[1-{e}^{-\frac{dis\times rati{o}_{cr}}{10\times v}}\right]\times weight(Q)\\  & = & {I}_{j}\times \left[1-{e}^{-\frac{dis\times rati{o}_{cr}}{10* v}}\right]\times (aircraftbareweight+100\\  &  & \times (load\;factor\times number\;of\;seats)+50\times seat)\end{array}$$

${I}_{j}$ is the emission coefficient of pollution j of aviation kerosene^28^. $weight(Q)$ is the total weight of the aircraft. ${M}_{fuel}$ is the fuel coefficient, ${M}_{ff}=\mathop{\prod }\limits_{i=1}^{n}\frac{{W}_{i}}{{W}_{i-1}}$ is a fuel weight proportionality coefficient, which is usually calculated by Fuel Percentage Method (FPM). The total sections of a whole flight contain seven task sections: Engine Starting, Taxiing, Taking Off, Climbing, Cruising, Descending and Landing. ${W}_{i}/{W}_{i-1}$ as the fuel weight proportionality coefficient of task section $i\;(i=1,2,\ldots ,7)$. $number\;of\;seats$ is certified seat number, $seat$ is the actual passenger number.

As we only consider the CCD section in this study, so we define the ${W}_{i}/{W}_{i-1}$ of other sections is 1. The ${W}_{i}/{W}_{i-1}$ of Climbing and Descending are 0.980 and 0.990. The equation of the CCD section to calculate ${W}_{i}/{W}_{i-1}$ is ${W}_{i}/{W}_{i-1}={e}^{-\frac{dis\times {c}_{cr}}{10\times v\times L{D}_{cr}}}$. *dis* is the cruising distance, $v$ is the cruising speed, ${c}_{cr}$ is the fuel consumption ratio when the aircraft is cruising, $L{D}_{cr}$ is the lift-drag ratio when the aircraft is cruising. The value of ${c}_{cr}$ and $L{D}_{cr}$ has direct relationships with the aircraft type. We define $rati{o}_{cr}=\frac{{c}_{cr}}{LD{c}_{cr}}$, and then for the cruising task section, the ${W}_{i}/{W}_{i-1}$is ${W}_{i}/{W}_{i-1}={e}^{-\frac{dis\times rati{o}_{cr}}{10* v}}$.

The actual flying time of each flight is applied to check the results of $rati{o}_{cr}$, and get the emission intensity.

For CO_2_, the emission coefficient is fixed, which is ${I}_{CO2}=3.157\;kg/kg$;

For SO_2_, the emission coefficient is fixed, which is ${I}_{SO2}=3.870\;g/kg$;

For CO and HC, ${I}_{j}={I}_{j0}\times \frac{{\theta }^{3.3}}{{\delta }^{1.02}}$. *θ* is the ratio of outside temperature to 288 K; *δ* is ratio of external pressure to sea level pressure. ${I}_{j0}$ is the standard emission coefficient of a LTO stage OF CO or HC (g/kg).

For NO_x_, ${I}_{NOx}={I}_{j0}\times \frac{{\delta }^{0.51}}{{\theta }^{1.65}}\times {\rm{\exp }}\left(19.0\times \left(0.0063-\frac{0.622\times \varphi \times Pv}{P-\varphi \times Pv}\right)\right)$. ${I}_{j0}$ is the standard emission coefficient of a LTO stage of NO_x_ (g/kg). *θ* is the ratio of outside temperature to 288 K; *δ* is ratio of external pressure to sea level pressure. *φ* is atmospheric relative humidity; *P* is external pressure; $Pv$ is atmospheric saturation pressure, which is calculated by Goff-Gratch formula^29^:

$$\begin{array}{ccc}lgPv & = & 10.79574\times \left(1-\frac{273.16}{T}\right)-5.02800\times lg\left(\frac{T}{273.16}\right)+1.50475\times 1{0}^{-4}\\  &  & \times \,\left[1-1{0}^{8.2969\times \left(1-\frac{T}{273.16}\right)}\right]+0.42873\times 1{0}^{-3}\times \left[1{0}^{4.76955\times \left(1-\frac{T}{273.16}\right)}\right]+0.78614\end{array}$$

According to relevant physical laws^29–31^, The external pressure *P* is ${\rm{P}}=101325\ast {\left(1-\frac{H}{44300}\right)}^{5.256}$. H is height. The outside temperature T is ${\rm{T}}=291.15-\frac{6\times H}{1000}$. The atmospheric relative humidity *φ* is $\varphi =100\times \frac{a\times (1+T/273.16)}{0.8\times Pv}$. *a* is absolute humidity, and it is ${\rm{a}}=\frac{26}{233211}\times {T}^{3}-\frac{302}{3731}\times {T}^{2}+\frac{569}{29}\times T-\frac{17461}{11}$.

For PM2.5, according to First Order Approximation (FOA) method^21^, it can be divided into Nonvolatile Component Fine Particles (NCFP) and Volatile Component Fine Particles (VCFP). For NCFP, ${I}_{NCFP}=0.054\times AFR\times {\left(SN\right)}^{1.234}+0.877$. The unit of ${I}_{NCFP}$ is mg/kg. $AFR$ is Air-Fuel Ratio, which is decided by height. *SN* is engine smoke, which can be found in the ICAO Aircraft Engine Emissions Databank^28^. For VCFP, it contains Volatile Organic Components (VOC) and Volatile Sulfur Components (VSC). For VOC, ${I}_{VOC}=\sigma \times {I}_{HC}$. *σ* is the ratio of VOC to the emission coefficient of HC, which can be found in ICAO Aircraft Engine Emissions Databank^28^. For VSC, ${I}_{VSC}=3\times 1{0}^{6}\times 0.2 \% \times 3.3 \%$. $0.2 \%$ is fuel sulfur content and $3.3 \%$ is sulfur conversion coefficient. Therefore, for PM2.5, ${I}_{PM2.5}={I}_{NCFP}+{I}_{VOC}+{I}_{VSC}$.

### ICAO standard method to calculate LTO emissions

The take-Off and Landing stage (LTO) refer to the aircraft’s whole process during takeoff and landing. This stage defined by ICAO includes four states, including approaching, taxiing, taking-off, and climbing, which defines climbing as the boundary layer from the end of aircraft takeoff to the aircraft’s flight out of the atmosphere. Therefore, this paper uses the standard LTO cycle definition specified by ICAO to calculate the fuel consumption, including all activities at an altitude below 3000 feet (915 m) near the airport. Therefore, this stage is not directly related to the route. In addition, the climbing process requires higher fuel consumption than the cruise phase at a constant altitude. Thus, the Climb, Cruise, and Descent cycle (CCD) are defined as all activities that occur at the height of 3000 feet (915 meters). Thus, fuel use accounts for most of the whole voyage and is directly related to the flight distance.

The calculation formula of the five non-CO_2_ pollution emissions in LTO stage is:

2 $${E}_{LTO}=\sum _{m}{P}_{a}\times {N}_{a}\times {C}_{m}\times {t}_{m}$$

${E}_{LTO}$ is the emissions in the LTO stage; ${P}_{a}$ is the standard emissions of the engine of aircraft type *a* (unit: kg); ${N}_{a}$ is the number of engines of aircraft type *a*; ${C}_{m}$ is the thrust setting of stage *m*; ${t}_{m}$ is the working time of phase *m*. The value range of *m* is 1, 2, 3, and 4, respectively corresponding to the four stages of takeoff and landing in the aircraft flight process: takeoff, climb, approach and taxiing. According to the standard LTO cycles defined by ICAO, when the aircraft is taking off, its engines are at 100% thrust and working time is 0.7 minutes; when the aircraft is climbing, its engines are at 85% thrust and working time is 2.2 minutes; when the aircraft is approaching, its engines are at 30% thrust and working time is 4 minutes; when the aircraft is taxiing, its engines are at 7% thrust and working time is 26 minutes. Therefore, in a standard LTO cycle, the total working time is 32.9 minutes.

The fuel consumption rate is calculated as:

3 $${F}_{am}=\frac{1}{A}\sum _{j}{K}_{j}{F}_{jmi}$$

*A* is the total number of airlines with aircraft type *a*; *j* is the type of engine of the aircraft; ${K}_{j}$ is the number of aircraft type *a* equipped with engine type *j*; ${F}_{jmi}$ is the fuel consumption rate of engine type *j* under the *m* setting. The data is from the ICAO Aircraft Engine Emissions Databank^28^. This formula is based on the weighted average of all possible engine types of the domestic routes in China.

## Supplementary information

Supplementary Table S1

Supplementary Table S2

Supplementary Table S3

Supplementary Table S4

Supplementary Table S5

Supplementary Table S6

Supplementary Table S7

Supplementary Table S8

Supplementary Table S9

Supplementary Table S10

Supplementary Table S11

Supplementary Table S12

## Data Availability

All the data on CCD emissions, LTO emissions, emission intensities is available as figshare datasets^32^.

## Code availability

The calculation of data is mainly done by Excel 2017, and there is no special code.
